# Supplementary material for: Metabolic reprogramming and membrane glycan remodeling as potential drivers of zebrafish heart regeneration
Source: Commun Biol. 2022 Dec 13;5:1365. doi: 10.1038/s42003-022-04328-2 (PMC9744865; doi:10.1038/s42003-022-04328-2)
Supplement: Supplementary file 2 — Supplementary Information [file 42003_2022_4328_MOESM2_ESM.pdf]

Supplementary information

Metabolic reprogramming and membrane glycan remodeling as potential drivers of zebrafish heart regeneration

Renza Spelat[1,2,#], Federico Ferro[1,3,#], Paolo Contessotto[1,4,#], Amal Samir Saad Soliman Aljaabary[1], Sergio M. Saldana[1], Chunsheng Jin[5], Niclas G. Karlsson[5], Maura Grealy[6], Markus M. Hilscher[7], Fulvio Magni[8], Clizia Chinello[8], Michelle Kilcoyne[1,9] and Abhay Pandit[1,]*

[1]CÚRAM, SFI Research Centre for Medical Devices, National University of Ireland Galway, Ireland

[2]Neurobiology sector, International School for Advanced Studies (SISSA), Trieste, Italy

[3]Department of Medical Surgery and Health Science, University of Trieste, Trieste, Italy

[4]Department of Molecular Medicine, University of Padova, Italy

[5]Department of Medical Biochemistry, Institute of Biomedicine, Sahlgrenska Academy, University of Gothenburg, Gothenburg, Sweden

[6]Pharmacology and Therapeutics, School of Medicine, National University of Ireland Galway, Ireland

[7]Science for Life Laboratory, Department of Biochemistry and Biophysics, Stockholm University, Stockholm, Sweden

[8]Clinical Proteomics and Metabolomics Unit, School of Medicine and Surgery, University of Milano-Bicocca, Vedano al Lambro, Italy

[9]Carbohydrate Signalling Group, Microbiology, School of Natural Sciences, National University of Ireland Galway, Galway, Ireland

[*]Corresponding author e-mail: abhay.pandit@nuigalway.ie

# These authors contributed equally

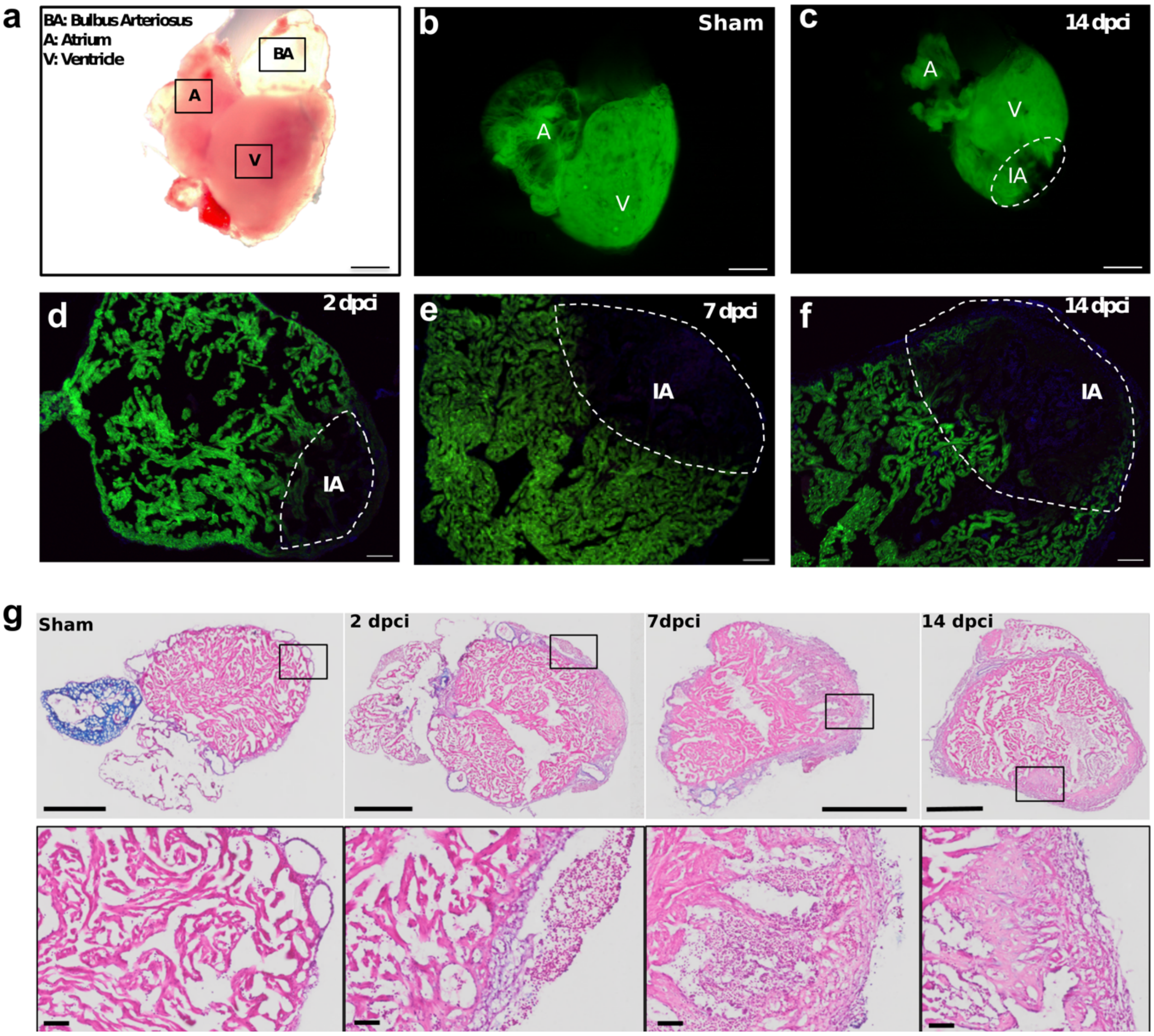

Supplementary Figure 1. Cryoinjury-induced myocardial infarction in zebrafish heart. a Whole-mount view of zebrafish heart showing the single atrium (A), ventricle (V) and the bulbus arteriosus (BA). **b** Whole uninjured (sham) and **c** 14 dpci hearts of transgenic zebrafish expressing GFP (Green Fluorescent Protein) under the control of cardiac specific promoter *cmlc-2* showing how the injured area (IA: encircled by dashed line) is devoid of GFP expression due to cardiomyocyte death. Scale bar 500 µm. This is more evident in the cross-sections shown in **d** 2 dpci, **e** 7 dpci and **f** 14 dpci where is also possible to see how cardiomyocytes are repopulating the injured area. Scale bar 100 µm. **g** Alcian blue staining in uninjured (sham) and cryo-injured (2 dpci, 7 dpci and 14 dpci) heart showing the fibrotic scar formation. Scale bar = 500 µm and 50 µm for the higher magnifications.

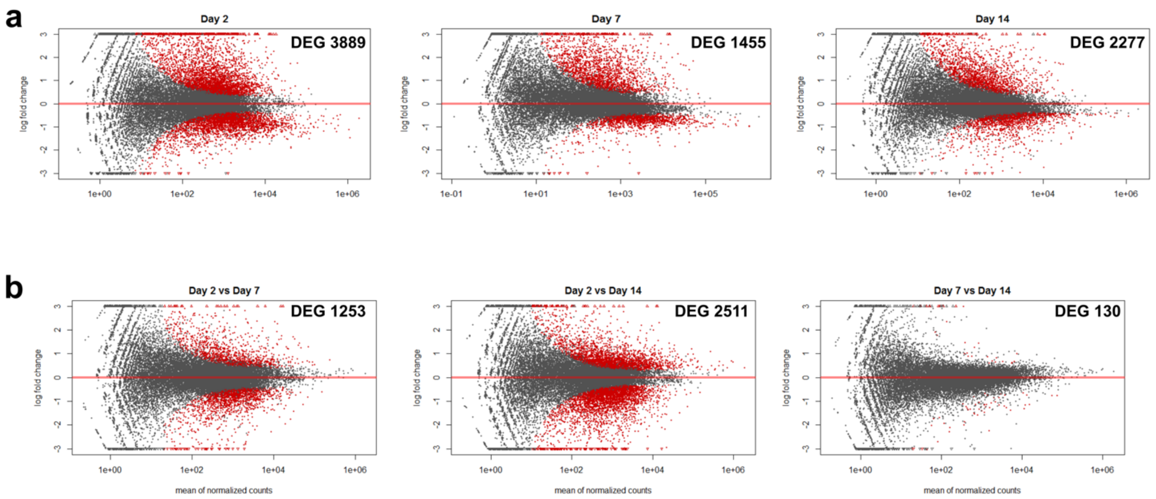

Supplementary Figure 2. RNA-sequencing analysis on regenerating zebrafish heart. a Volcano plots showing differentially expressed genes (DEG) at the different time points (2 days, 7 days and 14 days) after cryoinjury, and **b** 2 days vs 7 days, 2 days vs 14 days and 7 days vs 14 days. Red dots denote statistically significant DEG.

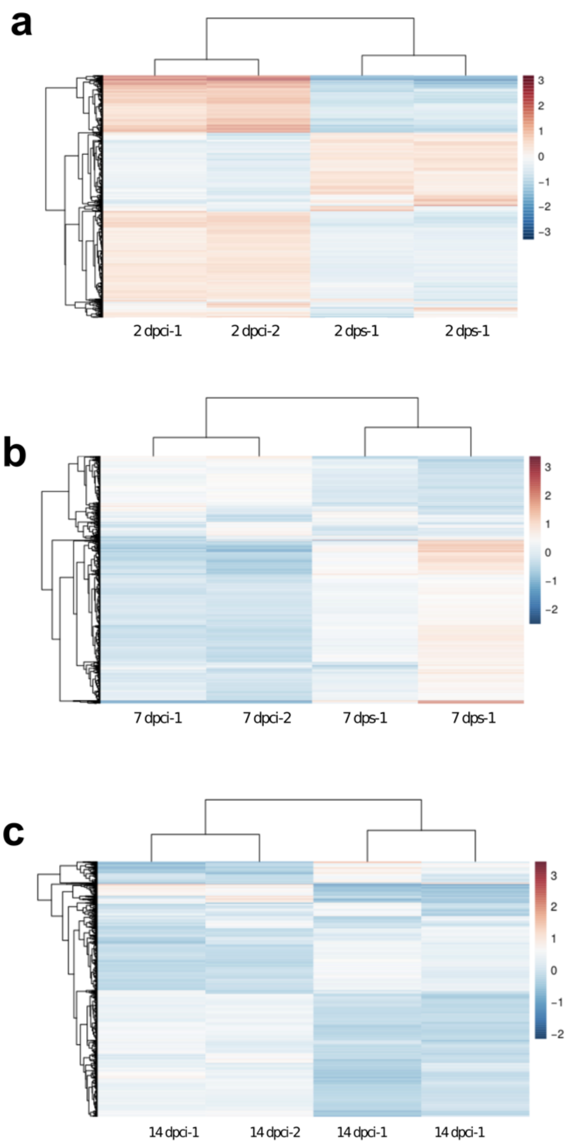

Supplementary Figure 3. Differentially expressed genes clustering. a Heatmap of the top 2000 differentially expressed genes (DEG) of the biological replicates 1 and 2 at 2 dpci, **b** 7 dpci and **c** 14 dpci compared with the corresponding controls dps (days post sham). DEG are located on each row, while columns represent the biological replicates. Cryoinjury replicates (dpci) cluster with each other, while the control replicates (dps) cluster together. Color intensity represents log_2 FC. Each biological replicate derives from the pooling of 4 different animals (n = 8).

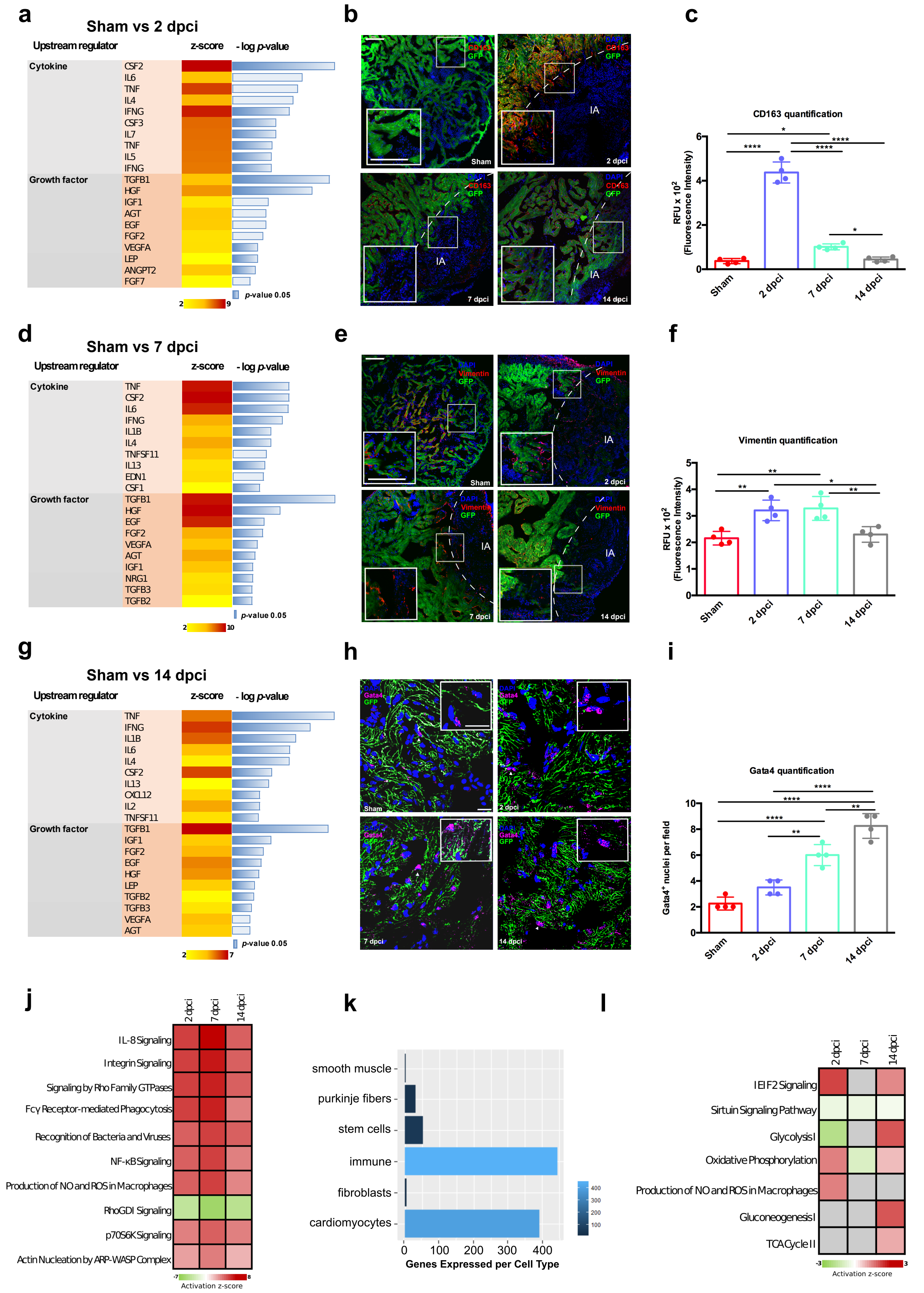

Supplementary Figure 4. Upstream regulators at the different phases of regeneration. a Upstream regulators at 2 dpci obtained analysing the RNA-sequencing data and using the IPA® URA tool. The most significant cytokine and growth factors are shown according to their

-log(*p*-value) and z-score. **b** Confocal images and **c** relative quantification showing CD163 expression in the Sham and following injury. Cardiomyocytes are identified by GFP positivity. Scale bar = 100 µm. **d** Upstream regulators at 7 dpci identified as described in **a**. **e** Confocal images and **f** relative quantification showing vimentin expression in the Sham and following injury. **g** Upstream regulators at 14 dpci identified as described in **a**. **h** Confocal images and **i** relative quantification showing Gata4 expression in the Sham and following injury. Scale bar = 100 µm. Scale bar = 10 µm. (*$p < 0.05$, * *$p < 0.01$, * * * $p < 0.001$, * * * * $p < 0.0001$). Data are shown as mean with SD (n = 4 animals per group). **j** Comparative analysis of the most significant pathways across the time points from RNA-seq data. **k** inference of cell type-proportions (RNA-seq deconvolution) obtained using the CellMapper package **l** Comparison analysis between the different time points of the most significant pathways identified using MS data. Two biological replicates deriving from the pooling of 4 different animals (n = 8 animals per group) were analysed for RNA-seq and MS experiments.

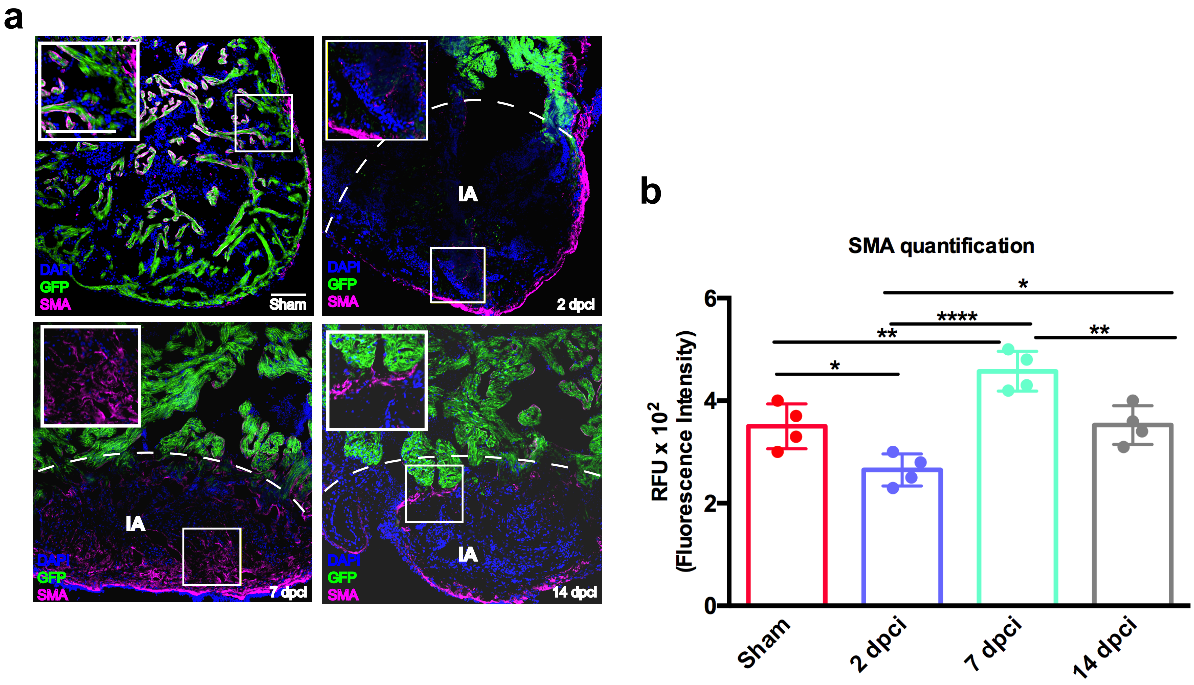

Supplementary Figure 5. Smooth muscle actin expression during zebrafish heart regeneration. a Confocal images and **b** relative quantification showing smooth muscle actin (SMA) expression in the Sham and following injury. Cardiomyocytes are identified by GFP positivity. Data are shown as mean with standard deviation (n = 4 animals per group). Scale bar = 100 µm. (*$p < 0.05$, * *p< 0.01, * * * $p < 0.001$, * * * * $p < 0.0001$).

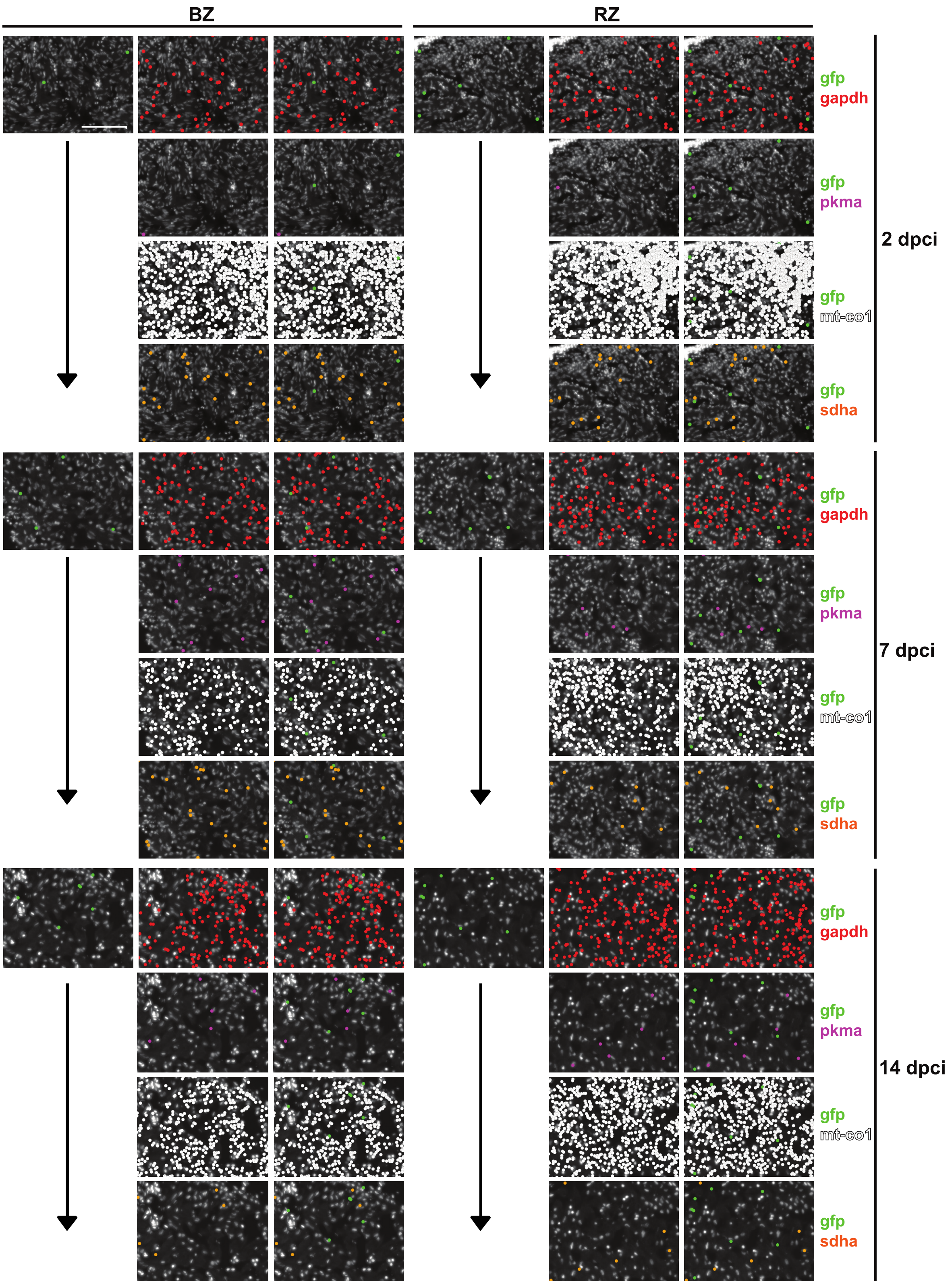

Supplementary Figure 6. Cardiomyocyte targeted in situ sequencing. Representative zoom in images of targeted multiplexed mRNA detection assay showing spatial distribution of spots identifying *gapdh*, *pkma*, *mt-co1* and *sdha* expression at 2, 7 and 14 dpci in the border and

remote zones. A transgenic zebrafish line, in which a green fluorescent protein (*gfp*) gene was inserted under the promoter of cardiomyocyte-specific gene cardiac myosin light chain 2 (*cmlc2*), was employed to identify cardiomyocytes. IA: injured area; BZ: border zone; RZ: remote zone. Scale bar bar = 100 µm. Two biological replicates for each group were analysed.

Supplementary Table 1 Most 25 significant GO terms from RNA-seq data at 2 dpci (days post cryoinjury).

ID	TERM	*p*Value
GO:0005975	carbohydrate metabolic process	1.15E-09
GO:0034314	Arp2/3 complex-mediated actin nucleation	1.22E-07
GO:0007229	integrin-mediated signaling pathway	2.57E-07
GO:0031101	fin regeneration	1.96E-04
GO:0006457	protein folding	2.07E-04
GO:0006508	proteolysis	3.27E-04
GO:0061077	chaperone-mediated protein folding	3.69E-04
GO:0030593	neutrophil chemotaxis	5.65E-04
GO:0051603	proteolysis involved in cellular protein catabolic process	8.84E-04
GO:0006487	protein N-linked glycosylation	0.00112
GO:0071028	nuclear mRNA surveillance	0.00146
GO:0030225	macrophage differentiation	0.00166
GO:0034475	U4 snRNA 3'-end processing	0.00472
GO:0048385	regulation of retinoic acid receptor signaling pathway	0.00472
GO:0009617	response to bacterium	0.00574
GO:0015992	proton transport	0.00574
GO:0070098	chemokine-mediated signaling pathway	0.00674
GO:0034427	nuclear-transcribed mRNA catabolic process	0.00725
GO:0030833	regulation of actin filament polymerization	0.00725
GO:0043420	anthranilate metabolic process	0.00850
GO:0019805	quinolinate biosynthetic process	0.00850
GO:0007188	adenylate receptor signaling pathway	0.01179
GO:0010501	RNA secondary structure unwinding	0.01377
GO:0006935	chemotaxis	0.01584
GO:0002430	complement receptor mediated signaling pathway	0.01639

Supplementary Table 2 Most 25 significant GO terms from RNA-seq data at 7 dpci.

ID	TERM	*p*Value
GO:0005975	carbohydrate metabolic process	2.55E+06
GO:0006954	inflammatory response	2.68E+09
GO:0006364	rRNA processing	2.70E+09
GO:0006457	protein folding	3.42E+10
GO:0002376	immune system process	7.37E+09
GO:0009617	response to bacterium	1.03E+10
GO:0034314	Arp2/3 complex-mediated actin nucleation	2.02E+11
GO:0015031	protein transport	2.41E+11
GO:0051603	proteolysis involved in cellular protein catabolic process	5.54E+11
GO:0042254	ribosome biogenesis	1.35E+12
GO:0006508	proteolysis	1.78E+12
GO:0006048	UDP-N-acetylglucosamine biosynthetic process	1.90E+12
GO:0045454	cell redox homeostasis	3.85E+12
GO:0030225	macrophage differentiation	6.19E+11
GO:0007229	integrin-mediated signaling pathway	6.78E+11
GO:0042127	regulation of cell proliferation	0.00115
GO:0045087	innate immune response	0.00117
GO:0031101	fin regeneration	0.00133
GO:0030833	regulation of actin filament polymerization	0.00146
GO:0030163	protein catabolic process	0.00224
GO:0035701	hematopoietic stem cell migration	0.00332
GO:0032496	response to lipopolysaccharide	0.00340
GO:0009611	response to wounding	0.00347
GO:0006487	protein N-linked glycosylation	0.00420
GO:0043410	positive regulation of MAPK cascade	0.00612

Supplementary Table 3 Most 25 significant GO terms from RNA-seq data at 14 dpci.

ID	TERM	*p*Value
GO:0006364	rRNA processing	1.15E-09
GO:0042254	ribosome biogenesis	1.22E-07
GO:0051603	proteolysis involved in cellular protein catabolic process	2.57E-07
GO:0006457	protein folding	1.96E-04
GO:0015031	protein transport	2.07E-04
GO:0000462	maturation of SSU-rRNA from tricistronic rRNA transcript	3.27E-04
GO:0009617	response to bacterium	3.69E-04
GO:0005975	carbohydrate metabolic process	5.65E-04
GO:0051028	mRNA transport	8.84E-04
GO:0000398	mRNA splicing, via spliceosome	0.00112
GO:0031101	fin regeneration	0.00146
GO:0006606	protein import into nucleus	0.00166
GO:0006406	mRNA export from nucleus	0.00472
GO:0043248	proteasome assembly	0.00472
GO:0045899	positive regulation of RNA polymerase II	0.00574
GO:0034314	Arp2/3 complex-mediated actin nucleation	0.00574
GO:0042127	regulation of cell proliferation	0.00674
GO:0042273	ribosomal large subunit biogenesis	0.00725
GO:0002376	immune system process	0.00725
GO:0006508	proteolysis	0.00850
GO:0010501	RNA secondary structure unwinding	0.00850
GO:0045454	cell redox homeostasis	0.01179
GO:0045087	innate immune response	0.01377
GO:0030833	regulation of actin filament polymerization	0.01584
GO:0006487	protein N-linked glycosylation	0.01639

Supplementary Table 4 Literature summary of the most recent publications with RNA-seq datasets in zebrafish' cryoinjury model and comparison with our results.

Aim	Model and/or fish strain	Early post-injury stage (from 0 to 6dpi)	Mid-term post injury (from 7dpi to 13pi)	Long-term remodelling (from 14 dpi)	Comparison with our results	Ref
Computational analysis of published ablation model datasets to select 15 candidate genes with potential role on heart regeneration; Comparison with a cryoinjured dataset at 5 timepoints: control, 1, 3, 5 and 7 day after operation	WIK wild-type fish	NA	NA	NA	Limited amount of data since only 15 candidate genes were analysed. The computational comparison showed concordance across two different models at 1 and 3 dpi, but there is no comparison between timepoints.	2014[1]
Generation and integration of a co-expression network of heart regeneration in zebrafish.	Wild type	Massive cell death from 4hpi; Lack of cardiomyocytes and fibrotic scar formation at 3dpi; Enrichment of genes implicated in energy metabolism, amino-acid biosynthesis and DNA replication, which may suggest cell proliferation,	Boost of regeneration at 7 dpi (enrichment in peptidase activity, DNA metabolism and replication enrichment)	No differences can be detected at 90 dpi with controls since remodelling is complete	As in our group, the largest numbers of differentially-expressed genes were detected at the early stages of regeneration. Authors performed a longer time-depended assess at 4 hours (hpi), 1, 3, 7, 14 and 90 dpi.	2016[2]

Study of collagen-producing cells subpopulations and their fate after zebrafish myocardium regeneration using different transgenic strains that present a genetic ablation of collagen producing cells	Tg(postnb:citrine)^{cn6}; Tg(periostin:CreER^{T2})^{cn7}; Tg(col1a2:loxPtagBFP-loxP-mCherry-NTR)^{cn8}; Tg(col1a2:loxP-mCherry-NTR)^{cn11}; Tg(fli1a:CreERT2)^{cn9}; Tg(kdrl:mcherry); Tg(-3.5ubb:loxP-loxP-eGFP)^{cz1702Tg} ; Tg(ubb:Switch)^{cz1701}; Tg(ubb:mCherry)^{cz1705Tg}; Tg(fli1a:GFP)^{y1};Tg1(-6.8wt1a:EGFP)^{li7Tg} and Tg(tcf21:CreERT2)^{pd42Tg}	NA	ECM genes upregulated at 7dpi	Decrease in ECM gene expression at 60 dpi<br>there were also collagen-encoding genes that were up-regulated at 60 dpi	Focused on ECM production and accumulation during zebrafish remodelling after cryoinjury.<br><br>Study of the gene expression at day 7 and 60 dpi, and comparison with the uninjured expression.<br><br>At 7dpi the upregulated pathways such as “Hepatic Fibrosis/Hepatic Stellate Cell Activation”, “Leukocyte Extravasation Signalling” or “Axonal Guidance Signalling”, are consistent with our data. However, authors sorted cell populations before the RNAseq analysis.	2018[3]

Dynamic transcriptome response monitoring of mRNA and microRNA in zebrafish at 1–160 dpi	Adult wild type	Increase of regeneration at 1 and 4dpi	Largest peaks of differentially expressed genes compared to controls were found at 4 and 7dpi.<br>Using GO gene sets, and enrichment in proliferation, in immune response and migration was reported.	From 14dpi an enrichment in DNA packaging, ECM organization and cell adhesion reported;<br><br>From 60dpi authors reported an enrichment in cardiovascular development, heart contraction and cell growth	Spatio-temporal resolution of the mRNAs during heart regeneration at different stages after injury. Focused on the identification of miRNA-mRNA interactions to described potential therapeutic targets for MI. Similar results related to cell proliferation enrichment during the first 2 weeks after the injury.<br>No linkage assessed within the metabolic stage and glycosylation during regeneration.	2018[4]

Single-cell RNA-seq analysis to explore the differential mechanisms between non-proliferating and proliferating cardiomyocytes	Tupfel Long Fin (TL); Tg(*myl7*:dsRED)$^{\text{s879Tg}}$; Tg(*myl7*:GFP)$^{\text{twu34Tg}}$; Tg(*phd3:*GFP)$^{\text{sh144}}$; Tg(*gata4:*EGFP)$^{\text{ae1}}$; TgBAC(*nppa:mCitrine)*<br><br>And<br><br>Tg(cmlc2:CreER)$^{\text{pd10}}$ x Tg(β-act2:BSNrg1)$^{\text{pd107}}$hearts; Tg(*gata4:*EGFP)$^{\text{ae1}}$hearts Tg(phd3:GFP)$^{\text{sh144}}$hearts	NA	NA	NA	Consistency with our results related to a metabolic shift in adult cardiomyocytes from OXPHOS to glycolysis during heart regeneration. From a mechanistic point of view, authors confirmed by blocking Nrg1/ErbB2 signaling, their pivotal role in the metabolic reprogramming.<br>No differential analysis and comparison performed over different timepoints after cryoinjury.	2019[5]
Study of cardiomyocyte proliferation after injury in adult zebrafish through metabolic reprogramming from a mechanistic point of view by the manipulation of pyruvate metabolism using Pdk3 as well as a catalytic subunit of the pyruvate dehydrogenase complex (PDC	*Tg(cryaa:DsRed,-5.1myl7:CreERT2)*^{pd10}; *pkma2*^{s717} ; *pkmb*^{s718} ; and *ppargc1a*^{bns176} ; *Tg(cryaa:Cerulean,hsp70l:LOXP-STOP-LOXP-pdha1aSTA-T2A-mCherry)*^{bns343}; *Tg(cryaa:Cerulean,hsp70l:LOXP-STOP-LOXP-pdk3b-T2A-mCherry)*^{bns344}	NA	NA	NA	Mechanistic analysis of glycolysis by Pdk3 which seems to promote cardiomyocyte dedifferentiation after injury. However only on timepoint at 5dpci was used.<br>Consistency in the results about the metabolic shift from OXPHOS to glycolysis. Authors reported a reduction in mitochondrial gene expression that	2020[6]

					indicates lower mitochondrial activity, and thereby reduced OXPHOS during regeneration.	
Study of the transcriptome during heart regeneration, whit a focus on the role of the suppression of transcription factor Tp53 associated genes.	Wild-type or transgenic zebrafish of the EK/AB strain *β-actin2:loxp-mCherry-STOP-loxp-DTA*^{pd36}; *β-actin2:loxp-mTagBFP-STOP-loxp-nrg1*^{pd107}; *β-actin2:loxp-mTagBFP-STOP-loxp-vegfaa*^{pd262}; *cmlc2:CreER*^{pd10}; *gata4:EGFP* ; *gata4:dsRed2*^{pd28} ; *tp53*^{M214K}	NA	High index of cardiomyocyte proliferation at 7dpi,<br><br>Dowstream of the Tp53 transcriptional network at 7dpi	High index of cardiomyocyte proliferation at 14dpi	Upstream regulation of factors involved in inflammation such as TNF-α and IL-6, as reported in our work. Results of earlier timepoints after injury not available. Mechanistic asses of Tp53 role in cardiomyocyte differentiation and division after cryoinjury by its suppression by mdm2	2020[7]
Study of macrophage response in zebrafish, as well as a comparison between neonatal and adult conditions of heart repair in mice proving their role in fibrosis and scar formation in the injured heart.	Wild type, *TgBAC(mpeg1:BirA-Citrine)*^{ox122}; *Tg(βactin:Avi-Cerulean-RanGap)*^{ct700a}; *TgBAC(mpeg1:BirA-Citrine;βactin:Avi-Cerulean-Rangap)*^{ox133}; *Gt(foxd3Citrine)*^{ct110}; *Tg(mpeg1:EGFP)*^{gl22}; *Tg(mpeg1:mCherry)*^{gl23}	Increased macrophage infiltration at 1dpi; genes realted to inflammatory response upregulated	From 5dpi upregulation of genes related to regeneration	Resolution of inflammation by 14dpi	The experimental design focused on the study of macrophages transcriptome at 1dpi, 5dpi and 14dpi, and the comparison with the resection model. Authors proved the role of macrophages in the scar formation and remodelling.	2020[8]

Supplementary Table 5 List of lectins printed on the microarray.

**Lectin source**	**Abbreviation**	**Binding specificity**
Artocarpus integrifolia	AIA	Gal, Gal-β-(1,3)-GalNAc
Robinia pseudoacacia	RPbAI	Gal, GalNAc
Sambucus nigra	SNA-II	Gal, GalNAc
Sophora japonica	SJA	β- GalNAc
Dolichos biflorus	DBA	GalNAc
Glechoma hederacea	GHA	GalNAc
Glycine max	SBA	GalNAc
Vicia villosa	VVA	GalNAc
Bauhinia purpurea	BPA	GalNAc/Gal
Wisteria floribunda	WFA	GalNAc/sulfated GalNAc
Helix pomatia	HPA	α-GalNAc
Griffonia simplicifolia	GSL-I-A4	α-GalNAc
Amarantus caudatus	ACA	Sialylated Gal-β-(1,3)-GalNAc
Agaricus bisporus	ABL	Gal-β-(1,3)-GalNAc, GlcNAc
Arachis hypogaea	PNA	Gal-β-(1,3)-GalNAc
Griffonia Simplicifolia	GSL-II	GlcNAc
Triticum vulgaris	sWGA	GlcNAc
Datura Stramonium	DSA	GlcNAc
Solanum tuberosum	STA	GlcNAc oligomers
Lycopersicum eculentum	LEL	GlcNAc- β-(1,4)- GlcNAc
Calystegia sepium	Calsepa	Man/Maltose
Narcissus pseudonarcissus	NPA	α-(1,6)-Man
Galanthus nivalis	GNA	Man-α-(1,3)-
Hyppeastrum Hybrid	HHA	Man-α-(1,3)- Man-α-(1,6)-
Canavalia ensiformis	ConA	α-linked Man, Glc, GlcNAc
Lens culinaris	Lch-B	Man, core fucosylated, agalactosylated biantennary N-glycans
Lens culinaris	Lch-A	Man/Glc
Pisum sativum	PSA	Man, core fucosylated trimannosyl N-glycans
Trichosanthes japonica	TJA-I	Sialic acid-α-(2-6)-Gal-β(1-4)-GlcNAc
Triticum vulgaris	WGA	NeuAc/GlcNAc
Maakia amurensis	MAA	Sialic acid-α-(2,3)-Gal(NAc)
Sambusus nigra	SNA-I	Sialic acid-α-(2,6)-Gal(NAc)
Cancer antennarius	CCA	O-acetylated sialic acids
Phaseolus vulgaris	PHA-L	tri-/tetra-antennary β-Gal/Gal- β-(1,4)-GlcNAc
Phaseolus vulgaris	PHA-E	biantennary, bisecting GlcNAc, β-Gal/Gal-β-(1,4)-GlcNAc
Ricinus communis	RCA-I/120	Gal-β-(1,4)-GlcNAc
Arum maculatum	AMA	Man
Cicer arietinum	CPA	Complex oligosaccharides
Caragana arborescens	CAA	Gal-β-(1,4)-GlcNAc
Erythrina cristagalli	ECA	Gal-β-(1,4)-GlcNAc oligomers
Trichosanthes japonica	TJA-II	Fuc-α-(1-2)-Gal
Aleuria aurantia	AAL	Fuc-α-(1,6)- and Fuc-α-(1,3)- linked
Lotus tetragonolobus	LTA	Fuc-α-(1,3)-, Fuc-α-(1,6)- and Fuc-α-(1,2)-linked
Ulex europaeus	UEA-I	Fuc-α-(1,2)-linked
Pseudomonas aeruginosa	PA-I	Gal, Gal derivates
Euonymous europaeus	EEA	Terminal α-linked Gal
Griffonia simplicifolia	GSL-I-B4	Terminal α-linked Gal
Maclura pomigera	MPA	Terminal α-linked Gal
Marasmius oreades	MOA	Terminal α-linked Gal

Supplementary references

1.Rodius, S. *et al.* Transcriptional response to cardiac injury in the zebrafish: systematic identification of genes with highly concordant activity across in vivo models. *BMC Genomics* **15**, 852 (2014).
2.Rodius, S. *et al.* Analysis of the dynamic co-expression network of heart regeneration in the zebrafish. *Sci Rep* **6**, 26822 (2016).
3.Sánchez-Iranzo, H. *et al.* Transient fibrosis resolves via fibroblast inactivation in the regenerating zebrafish heart. *Proc Natl Acad Sci U S A* **115**, 4188–4193 (2018).
4.Klett, H. *et al.* Delineating the Dynamic Transcriptome Response of mRNA and microRNA during Zebrafish Heart Regeneration. *Biomolecules* **9**, (2018).
5.Honkoop, H. *et al.* Single-cell analysis uncovers that metabolic reprogramming by ErbB2 signaling is essential for cardiomyocyte proliferation in the regenerating heart. *Elife* **8**, (2019).
6.Fukuda, R. *et al.* Stimulation of glycolysis promotes cardiomyocyte proliferation after injury in adult zebrafish. *EMBO Rep* **21**, e49752 (2020).
7.Shoffner, A., Cigliola, V., Lee, N., Ou, J. & Poss, K. D. Tp53 Suppression Promotes Cardiomyocyte Proliferation during Zebrafish Heart Regeneration. *Cell Rep* **32**, 108089 (2020).
8.Simões, F. C. *et al.* Macrophages directly contribute collagen to scar formation during zebrafish heart regeneration and mouse heart repair. *Nat Commun* **11**, 600 (2020).
