# Supplementary material for: Metabolic reprogramming and membrane glycan remodeling as potential drivers of zebrafish heart regeneration
Source: Commun Biol. 2022 Dec 13;5:1365. doi: 10.1038/s42003-022-04328-2 (PMC9744865; doi:10.1038/s42003-022-04328-2)
Supplement: Supplementary file 3 — Description of Additional Supplementary Files [file 42003_2022_4328_MOESM3_ESM.pdf]

# Description of Additional Supplementary Files

**File name:** Supplementary Data 1
**Description:** Differentially expressed genes (DEG) at 2 dpci (days post cryoinjury).

**File name:** Supplementary Data 2
**Description:** Differentially expressed genes (DEG) at 7 dpci (days post cryoinjury).

**File name:** Supplementary Data 3
**Description:** Differentially expressed genes (DEG) at 14 dpci (days post cryoinjury).

**File name:** Supplementary Data 4
**Description:** Proteins identified by nLC-ESI MS/MS label-free quantification.

**File name:** Supplementary Data 5
**Description:** Lectin microarray analysis of 2 dpci (days post cryoinjury) respect to the Sham.

**File name:** Supplementary Data 6
**Description:** Lectin microarray analysis of 7 dpci (days post cryoinjury) respect to the Sham.

**File name:** Supplementary Data 7
**Description:** Lectin microarray analysis of 14 dpci (days post cryoinjury) respect to the Sham.

**File name:** Supplementary Data 8
**Description:** Identified N-glycan structures.

**File name:** Supplementary Data 9
**Description:** Identified O-glycan structures.
