# Supplementary material for: Metabolic reprogramming and membrane glycan remodeling as potential drivers of zebrafish heart regeneration
Source: Commun Biol. 2022 Dec 13;5:1365. doi: 10.1038/s42003-022-04328-2 (PMC9744865; doi:10.1038/s42003-022-04328-2)
Supplement: Supplementary file 13 — Reporting Summary [file 42003_2022_4328_MOESM13_ESM.pdf]

nature portfolio

Corresponding author(s): Abhay Pandit

Last updated by author(s): Nov 27, 2022

Reporting Summary

Nature Portfolio wishes to improve the reproducibility of the work that we publish. This form provides structure for consistency and transparency in reporting. For further information on Nature Portfolio policies, see our Editorial Policies and the Editorial Policy Checklist.

Statistics

For all statistical analyses, confirm that the following items are present in the figure legend, table legend, main text, or Methods section.

n/a	Confirmed	
☐	☒	The exact sample size (*n*) for each experimental group/condition, given as a discrete number and unit of measurement
☐	☒	A statement on whether measurements were taken from distinct samples or whether the same sample was measured repeatedly
☐	☒	The statistical test(s) used AND whether they are one- or two-sided<br>*Only common tests should be described solely by name; describe more complex techniques in the Methods section.*
☒	☐	A description of all covariates tested
☐	☒	A description of any assumptions or corrections, such as tests of normality and adjustment for multiple comparisons
☐	☒	A full description of the statistical parameters including central tendency (e.g. means) or other basic estimates (e.g. regression coefficient) AND variation (e.g. standard deviation) or associated estimates of uncertainty (e.g. confidence intervals)
☐	☒	For null hypothesis testing, the test statistic (e.g. *F*, *t*, *r*) with confidence intervals, effect sizes, degrees of freedom and *P* value noted<br>*Give P values as exact values whenever suitable.*
☒	☐	For Bayesian analysis, information on the choice of priors and Markov chain Monte Carlo settings
☐	☒	For hierarchical and complex designs, identification of the appropriate level for tests and full reporting of outcomes
☒	☐	Estimates of effect sizes (e.g. Cohen's *d*, Pearson's *r*), indicating how they were calculated

Our web collection on statistics for biologists contains articles on many of the points above.

Software and code

Policy information about availability of computer code

Data collection	Data acquisition and processing for LC-ESI-MS/MS analysis of glycans were conducted using Xcalibur™ software (Version 2.0.7, Thermo Fisher Scientific, Waltham, MA, USA). Data collection and elaboration of proteomic analysis by nLC-ESI MS/MS was performed through DataAnalysis™ v.4.1 Sp4 (Bruker Daltonics, Germany).
Data analysis	IPA® software (Qiagen, Redwood City, CA, www.qiagen.com/ingenuity) was used to analyse RNA- sequencing data. For imaging and quantification of immunofluorescence ImageJ v1.51w software (Rasband, W.S., ImageJ, U. S. National Institutes of Health, Bethesda, Maryland, USA) was used. Clustering analysis was conducted using Clustering Explorer v3.5 (HCE 3.5, University of Maryland, http:// www.cs.umd.edu/ hcil/hce/hce3.html). GO enrichment analysis for RNA-sequencing data was carried out using Database for Annotation, Visualization, and Integrated Discovery (DAVID, http://david.abcc.ncifcrf.gov). The R package GOplot (available via CRAN-The Comprehensive R Archive Network: http://cran.r-project.org/web/packages/GOplot) was used to visualize and analyse Gene Ontology data. Data collection and elaboration of proteomic analysis by nLC-ESI MS/MS was performed through DataAnalysis™ v.4.1 Sp4 (Bruker Daltonics, Germany). All statistical analyses were conducted using GraphPad Prism 6 (San Diego, CA). Targeted in situ sequencing was performed by following the pipelines available at https://github.com/Moldia.

For manuscripts utilizing custom algorithms or software that are central to the research but not yet described in published literature, software must be made available to editors and reviewers. We strongly encourage code deposition in a community repository (e.g. GitHub). See the Nature Portfolio guidelines for submitting code & software for further information.

Data

Policy information about availability of data

All manuscripts must include a data availability statement. This statement should provide the following information, where applicable:

- Accession codes, unique identifiers, or web links for publicly available datasets
- A description of any restrictions on data availability
- For clinical datasets or third party data, please ensure that the statement adheres to our policy

All the annotated MS/MS spectra of identified N-glycan and O-glycan structures are available in unicarb-DR (https://unicarb-dr.glycosmos.org/references/519). Raw glycomic data files are available at https://glycopost.glycosmos.org/entry/GPST000053. The raw RNA-sequencing data generated during the current study are available in the Genome Sequence Archive (National Genomics Data Center, China National Center for Bioinformation, Beijing Institute of Genomics, Chinese Academy of Sciences) with the accession number GSA: CRA009013, and are publicly accessible at https://ngdc.cncb.ac.cn/gsa. All data of proteins identified with nano-LC MS/MS are deposited in MassIVE and are available at https://massive.ucsd.edu/ProteoSAFe/dataset.jsp?task=661d66ff2c514775a3f052e550eaf498. All data that support the findings of this study are available from the corresponding author upon reasonable request.

Human research participants

Policy information about studies involving human research participants and Sex and Gender in Research.

Reporting on sex and gender	*Use the terms sex (biological attribute) and gender (shaped by social and cultural circumstances) carefully in order to avoid confusing both terms. Indicate if findings apply to only one sex or gender; describe whether sex and gender were considered in study design whether sex and/or gender was determined based on self-reporting or assigned and methods used. Provide in the source data disaggregated sex and gender data where this information has been collected, and consent has been obtained for sharing of individual-level data; provide overall numbers in this Reporting Summary. Please state if this information has not been collected. Report sex- and gender-based analyses where performed, justify reasons for lack of sex- and gender-based analysis.*
Population characteristics	*Describe the covariate-relevant population characteristics of the human research participants (e.g. age, genotypic information, past and current diagnosis and treatment categories). If you filled out the behavioural & social sciences study design questions and have nothing to add here, write "See above."*
Recruitment	*Describe how participants were recruited. Outline any potential self-selection bias or other biases that may be present and how these are likely to impact results.*
Ethics oversight	*Identify the organization(s) that approved the study protocol.*

Note that full information on the approval of the study protocol must also be provided in the manuscript.

Field-specific reporting

Please select the one below that is the best fit for your research. If you are not sure, read the appropriate sections before making your selection.

☒ Life sciences ☐ Behavioural & social sciences ☐ Ecological, evolutionary & environmental sciences

For a reference copy of the document with all sections, see nature.com/documents/nr-reporting-summary-flat.pdf

Life sciences study design

All studies must disclose on these points even when the disclosure is negative.

Sample size	Sample size was determined using the Power Analysis method.
Data exclusions	No data were excluded from the analyses.
Replication	The reproducibility of the experimental findings was successfully confirmed by means of technical and biological replicates.
Randomization	Animals and samples were randomly assigned to the different groups.
Blinding	Investigators were blinded to group allocation during data collection and analysis.

Reporting for specific materials, systems and methods

We require information from authors about some types of materials, experimental systems and methods used in many studies. Here, indicate whether each material, system or method listed is relevant to your study. If you are not sure if a list item applies to your research, read the appropriate section before selecting a response.

Materials & experimental systems

n/a	Involved in the study
☐	☒ Antibodies
☒	☐ Eukaryotic cell lines
☒	☐ Palaeontology and archaeology
☐	☒ Animals and other organisms
☒	☐ Clinical data
☒	☐ Dual use research of concern

Methods

n/a	Involved in the study
☒	☐ ChIP-seq
☒	☐ Flow cytometry
☒	☐ MRI-based neuroimaging

Antibodies

Antibodies used	The following primary antibodies were used: rabbit polyclonal to CD163 (ab87099, Abcam, UK), mouse monoclonal to vimentin (RV202, Abcam, UK), rabbit polyclonal to Gata 4 (ab61170, Abcam, UK) and rabbit polyclonal to α-smooth muscle actin (ab5694, Abcam, UK).
Validation	All the primary antibodies used were tested for the specificity in Danio rerio and for immunofluorescence application by the manufacture. The following dilutions were chosen after testing different concentrations: rabbit polyclonal to CD163 at 1:200, mouse monoclonal to vimentin and rabbit polyclonal to Gata 4 at 1:100, rabbit polyclonal to α-smooth muscle actin at 1:250.

Animals and other research organisms

Policy information about studies involving animals; ARRIVE guidelines recommended for reporting animal research, and Sex and Gender in Research

Laboratory animals	Danio rerio wild-type AB strain and transgenic strain cmlc2::GFP, both male and female aged 6-18 months were used in this study.
Wild animals	NA
Reporting on sex	Danio rerio samples were employed by using both male and females.
Field-collected samples	NA
Ethics oversight	All animal-related protocols were performed in accordance with national guidelines and approved by the Animal Care Research Ethics Committee (ACREC) at the National University of Ireland, Galway, and the Health Product Regulatory Authority (HPRA), Ireland.

Note that full information on the approval of the study protocol must also be provided in the manuscript.
