# Supplementary material for: Metabolic reprogramming and membrane glycan remodeling as potential drivers of zebrafish heart regeneration
Source: Commun Biol. 2022 Dec 13;5:1365. doi: 10.1038/s42003-022-04328-2 (PMC9744865; doi:10.1038/s42003-022-04328-2)
Supplement: Supplementary file 14 — Final Revisions Checklist [file 42003_2022_4328_MOESM14_ESM.docx]

***To the Author****— Please review the editorial comments and requests below and confirm that changes have been made in the manuscript in the right-hand column.* ***This document*** ***must be uploaded*** *as a related manuscript file.*

Please see our [final file submission checklist](https://www.nature.com/documents/commsj-file-checklist.pdf) for information about submitting your revised documents.

| **Files and General Policies** | |
| --- | --- |
| **Main manuscript file must be in Microsoft Word or LaTeX format.**  LaTex and Tex article source files must be accompanied by the compiled PDF for reference. The bibliography must be submitted separately (as a .bib file) or contained within the .tex file. |  |
| **Each Figure must be provided as a separate file** and must be supplied whole, with all panels included in a single document. Figures should be provided at a minimum resolution of 300 dpi at final size.  Figure files must only contain images (please also leave out labels such as “Figure 1” etc). Figure captions must instead be included within the main manuscript file, grouped together at the end of the document. | We have provided each Figure as a PDF separate files. |
| All figures, tables, and supplementary items must be cited in the manuscript and **numbered in the order in which they appear**. | We have revised Figure 1, 3 and 4, their legends and the relative “Results” section to have Figure panels citation in the manuscript in the same order they appear. |
| Please check whether your manuscript contains **third-party images**, such as figures from the literature, stock photos, clip art or commercial satellite and map data. We strongly discourage the use or adaptation of previously published images, but if this is unavoidable, please request the necessary rights documentation to re-use such material from the relevant copyright holders and return this to us when you submit your revised manuscript.  An appropriate permissions statement must be present in the relative figure caption for any third-party images. |  |
| **Please check that you have not copied any text directly from published work** (even your own) without clear attribution, including one or more references. We run a plagiarism detection software and may need to request additional changes if we identify large blocks of identical text. |  |
| An updated **editorial policy checklist** that verifies compliance with all required editorial policies must be completed and uploaded with the revised manuscript. All points on the policy checklist must be addressed; if needed, please revise your manuscript in response to these points.  <https://www.nature.com/documents/nr-editorial-policy-checklist.pdf>.  Please note that this form is a dynamic ‘smart pdf’ and must therefore be downloaded and completed in Adobe Reader. This file will not open in an internet browser. |  |
| The **reporting summary** will be published alongside your manuscript therefore it needs to accurately represent your work. In this case, please take a closer look at the reporting summary and make sure things are completed correctly. If an item does not apply, for example human participants, I need you to check the NA box next to that item. No section should be left blank.  Also, please make sure to include your name and date at the top of the document.  If you require a new Reporting Summary form, please download it here: <https://www.nature.com/documents/nr-reporting-summary.pdf>.  Please note that this form is a dynamic ‘smart pdf’ and must therefore be downloaded and completed in Adobe Reader. This file will not open in an internet browser. |  |
| Your paper will be accompanied by a brief editor's summary when it is published on our homepage. Please approve the draft summary below or provide us with a suitably edited version (no more than 250 characters including spaces).  **Transcriptomic and proteomic analyses reveal that glycosylation and carbohydrate metabolism are central processes in heart regeneration in zebrafish, including a shift from OXPHOS to glycolysis seven days after injury.** | We approve the summary proposed. |
| **ORCID**  *Communications Biology* is committed to improving transparency in authorship. As part of our efforts in this direction, we are now requesting that all authors identified as ‘corresponding author’ create and link their Open Researcher and Contributor Identifier (ORCID) with their account on the Manuscript Tracking System (MTS) prior to acceptance. ORCID helps the scientific community achieve unambiguous attribution of all scholarly contributions. For more information please visit <http://www.springernature.com/orcid>.  For all corresponding authors listed on the manuscript, please follow the instructions in the link below to link your ORCID to your account on our MTS before submitting the final version of the manuscript. If you do not yet have an ORCID you will be able to create one in minutes.  <https://www.springernature.com/gp/researchers/orcid/orcid-for-nature-research>  IMPORTANT: All authors identified as ‘corresponding author’ on the manuscript must follow these instructions. Non-corresponding authors do not have to link their ORCIDs but are encouraged to do so. Please note that it will not be possible to add/modify ORCIDs at proof. Thus, if they wish to have their ORCID added to the paper they must also follow the above procedure prior to acceptance.  To support ORCID's aims, we only allow a single ORCID identifier to be attached to one account. If you have any issues attaching an ORCID identifier to your MTS account, please contact the Platform Support Helpdesk at <http://platformsupport.nature.com/> |  |
| We regularly highlight papers published in *Communications Biology* on the journal’s **Twitter** account (@CommsBio). If you would like us to mention authors, institutions, or lab groups in these tweets, please provide the relevant twitter handles in the right-hand column. |  |
| We would welcome the submission of material for the **‘Featured Image’** section on the Communications Biology home page. Images should relate to the content of your manuscript but need not be contained within the paper. **Photographs and aesthetically interesting images are preferred; diagrams are generally not used.** Suggestions should be uploaded as a Related Manuscript file. Please provide 1200x675-pixel RGB images. You will also need to submit a completed [Image License to Publish](https://www.nature.com/documents/snl-image-ltp.docx).  Unfortunately, we cannot promise that your suggestions will be used. |  |
| **Supplementary information** | |
| **Supplementary Information Format and referencing**   - Supplementary Figures, small Tables, and any supplementary text must be provided **in a single PDF**. Figures and their captions should be presented together.   - If you include a title page, please check that the title and author list matches the main manuscript. - **All Supplementary items must be referred to in the manuscript**, and items must be mentioned in numerical order. Please do not include general references to “Supplementary Material”; instead refer to specific items. - Additional files can be provided as **Supplementary Data** (Excel files, text files, .zip folders), **Supplementary Movies**, **Supplementary Audio**, or **Supplementary Software** (.zip folder)   Supplementary Information files will be uploaded with the published article as they are submitted with the final version of your manuscript. Any highlighting or tracked changes should be removed from the file. | Please provide all tables and label the Tables in the SI as 1,2,3…, and the Supplementary Data tables separately (not part of the SI file).  We have numbered Supplementary Tables and Data accordingly. Supplementary Data files are provided separately and referred in the manuscript. |
| **Supplementary items must be cited in a consistent format**. Names of items in the Supplementary file(s) must match those used in the main manuscript.  **We recommend using the following naming formats:** Supplementary Figure 1, Supplementary Table 1, Supplementary Data 1, Supplementary Note 1, and Supplementary References. |  |
| **Large tables and other data types:** We strongly recommend depositing these to suitable repositories (such as Figshare, Dryad, or a data type-specific repository if one exists).  Otherwise, these must be supplied as **Supplementary Data** files. Each file must be labelled as Supplementary Data 1, etc. |  |

| **It’s mandatory to provide access to the numerical source data for graphs and charts:** We strongly recommend depositing these to suitable repositories (such as Figshare, Dryad, or a data type-specific repository if one exists).  Otherwise, all source data underlying the graphs and charts presented in the main figures must be uploaded as **Supplementary Data** (in Excel or text format). Note that only the data used directly for generating the charts needs to be supplied. | All source data for graphs presented in the main figures are provided in Supplementary Data 1-9 |
| --- | --- |

| For any Supplementary Files such as those mentioned above that are not included your combined PDF (e.g. Supplementary Data, Movies, Audio, Software), please provide a title and description for each file **here in the column to the right**.  For example:  File name: Supplementary Data 1  Description: The source data behind the graphs in the paper | Supplementary files not included in the combined PDF are:  File name: **Supplementary Data 1**  Description: The source data behind graphs of RNA seq in sham vs 2 dpci (days post cryoinjury) samples  File name: **Supplementary Data 2**  Description: The source data behind graphs of RNA seq in sham vs 7 dpci (days post cryoinjury) samples  File name: **Supplementary Data 3**  Description: The source data behind graphs of RNA seq in sham vs 14 dpci (days post cryoinjury) samples  File name: **Supplementary Data 4**  Description: The source data behind graphs of proteomic analysis with nLC-ESI MS/ at 2, 7, 14 dpci and sham samples  File name: **Supplementary Data 5**  Description: The source data behind graphs of lectin microarray in sham vs 2 dpci samples  File name: **Supplementary Data 6**  Description: The source data behind graphs of lectin microarray in sham vs 7 dpci samples  File name: **Supplementary Data 7**  Description: The source data behind graphs of lectin microarray in sham vs 14 dpci samples  File name: **Supplementary Data 8**  Description: identified N-glycans structures in 2, 7, 14 dpci and sham samples  File name: **Supplementary Data 9**  Description: identified O-glycans structures in 2, 7, 14 dpci and sham samples |
| --- | --- |
| **Supplementary References** should appear at the end of the Supplementary Information file. Numbering must start from 1.  If a supplementary reference also appears in the main manuscript reference list, please repeat it in the Supplementary References. |  |
| **Title Page** | |
| Please ensure that the author list provided in our manuscript tracking system matches the author list in the main manuscript. |  |
| **Please check that your author list and affiliations comply with the following:**   - Affiliations must be numbered in the order of their first appearance in the author list. - Each affiliation must contain only one address. - Each affiliation must include the institution, city and country. The name of the city must be provided separately from the institution even if it is a part of the institution name; e.g. ‘University of Science and Technology Beijing, Beijing, China’ - Author tagging statements are limited to the following two options: "These authors contributed equally" and "These authors jointly supervised this work", with no more than one of each tag permitted. - Where relevant, “present address” must be provided separately as the final affiliation. - At least one corresponding author must be designated, and an e-mail address must be provided for each corresponding author (with a limit of one e-mail address per author). | Please use numbers instead of letters.  Letters were substituted with numbers and  for equal contribution the statement  "These authors contributed equally" was used in the Main manuscript and Supplementary Information files. |
| **Manuscript title**  Please ensure the title clearly describes the central finding of the paper. We recommend writing the title as a declarative statement of approximately 15 words or fewer.  Be sure to include any key species, protein names, or gene names to ensure optimal retrieval of the paper in database searches. |  |
| **Main text** | |
| **Format of the main text**  Please ensure your manuscript includes the following sections, presented in this order:   1. “**Introduction”**: The background and rationale for the work. The final paragraph should be a brief summary of the major results and conclusions. The results of the current study must only be discussed in this final paragraph. The Introduction should contain no references to figures or tables. Do not include subheadings. 2. “**Results” or “Results and Discussion”**. This should be split into subheaded sections; we recommend 1 subheading per main figure or table. Figures should not be embedded in the text but submitted separately.    1. Do not use more than 1 layer of subheadings.    2. A “Conclusions” paragraph can be included **only if the results and discussion are combined into a single section.** 3. “**Discussion” (optional)**, without subheadings. 4. **Methods**, which should be split into subheaded sections. Do not use more than 1 layer of subheadings.   **To improve readability**, we recommend that the main text (Introduction, Results and Discussion) be limited to approximately 5000 words or fewer. |  |
| **Statistical reporting**  Wherever statistics have been derived (e.g. error bars, box plots, statistical significance) the legend needs to provide and define the n number (i.e. the sample size used to derive statistics) as a precise value (not a range), using the wording “n=X biologically independent samples/animals/independent experiments” etc. as applicable. |  |
| Language such as “new”, “novel”, “for the first time”, “unprecedented”, etc, should be avoided, or qualified with “**to the best of our knowledge**” or similar, because it often leads to unproductive controversy. **Novelty should be made clear from the context.** | We have qualified with “to the best of our knowledge” the following sentence (page 17, lines 398-399):  “Interestingly, here we observed for the first time to the best of our knowledge that, after the metabolic shift to glycolysis in the BZ” |
| **Display items** | |
| **Figure captions/legends**  Figures must have a title that will appear above the Figure **and** a legend that will appear below the Figure (see e.g. <https://www.nature.com/articles/s42003-020-1059-1/figures/1>)  The Figure title must describe the Figure as a whole and must not contain reference to specific figure panels.  The Figure legend must refer to and describe **all panels**. Abbreviations, symbols, colors, and shading present in the Figure must be defined. Please write out the symbols/colors in words (blue circles, red dashed line, etc.) within these definitions.  **All figure panels must be labelled using lower case letters. Please refrain from referring to sections of figures as top/bottom/left/right/, etc.** |  |
| **Axis and panel labels will be published as received.** We recommend using a sans-serif font such as Arial or Helvetica. |  |
| Please pay close attention to our [Digital Image Integrity Guidelines](https://www.nature.com/nature-research/editorial-policies/image-integrity). Also ensure that you retain unprocessed data and metadata files after publication, ideally archiving data in perpetuity, as these may be requested during the peer review and production process or after publication if any issues arise. |  |
| **Methods** | |
| Please ensure that all information present in the Reporting Summary is also in the manuscript. This information is usually most appropriate in the Methods section. |  |
| **We allow unlimited space for Methods.** The Methods must contain sufficient detail such that the work could be repeated. It is preferable that all key methods be included in the main manuscript, rather than in the Supplementary Information.  **Please avoid use of “as described previously”** or similar, and instead detail the specific methods used with appropriate attribution. | We have additionally detailed specific methods which were previously referred **“as previously described”** in the “Methods” section, in particular:  “Lectin microarrays containing 49 immobilized lectins (Supplementary Table 5) were utilized to give specific information on the repertoire of glycans present in the sample” (page 27, lines 630-631)  “Samples were incubated on lectin microarrays for 1 hour at room temperature” (page 27, lines 636-637)  “Samples were desalted with 25 μL of AG50WX8 cation-exchange resin (Bio-Rad, Hercules, CA, USA) and dried in a Savant SpeedVac” (page 30, lines 695-697)  “Membrane proteins were diluted to 1 mg/ml in 100 mM sodium bicarbonate, pH 8.2, and all following steps were carried out in the dark. Specifically, 1 mg of each sample was incubated with 50 μg of Alexa Fluor® 555 succinimidyl ester (Thermo Fisher Scientific, USA) dissolved in dry dimethyl sulfoxide” (page 27, lines 619-622) |
| **The Methods should include a separate section titled “Statistics and Reproducibility”** with general information on how the statistical analyses of the data were conducted, and general information on the reproducibility of experiments (also those lacking statistical analysis), including the sample sizes and number of replicates and how replicates were defined. | Please rename.  The section title has been renamed **“Statistics and Reproducibility”** |
| For studies using **live vertebrates**, a statement affirming that you have complied with all relevant ethical regulations for animal testing and research is necessary. A statement explicitly confirming if the study received ethical approval, including the name of the board and institution that approved the study protocol is also required. The species, strain, sex and age of animals should be included. |  |
| **Data Policies** | |
| The **Data Availability** **statement** must include:   - Access details for deposited data, including repository name and unique data ID. - How source data can be obtained. - A statement that all other data are available from the corresponding author (or other sources, as applicable) on reasonable request. **Note that ‘available upon request’ is only appropriate if immediate data access has not been mandated by our policies or by the editors.**   See here for more information about formatting your Data Availability Statement: <http://www.springernature.com/gp/authors/research-data-policy/data-availability-statements/12330880> | Please provide the RNA-seq acc number and refer to the Supplementary Data files in the DAS.  RNA- seq fastq files have been deposited in Genome Sequence Archive (GSA) and the relative acc number has been provided in the DAS:  “The raw RNA-sequencing data generated during the current study are available in the Genome Sequence Archive (National Genomics Data Center, China National Center for Bioinformation, Beijing Institute of Genomics, Chinese Academy of Sciences) with the accession number GSA: CRA009013, and are publicly accessible at <https://ngdc.cncb.ac.cn/gsa>.”  All data of proteins identified with nano-LC MS/MS are deposited in MassIVE and are available at <https://massive.ucsd.edu/ProteoSAFe/dataset.jsp?task=661d66ff2c514775a3f052e550eaf498>. |
| **Mandatory deposition** of raw and processed data is required for:   - All sequencing data (DNA, RNA, protein) - Novel human genetic polymorphisms (e.g., [dbSNP](https://www.ncbi.nlm.nih.gov/snp/)) - Linked genotype and phenotype data (e.g., [dbGaP](https://www.ncbi.nlm.nih.gov/gap/) for human data) - GWAS summary statistics or polygenic risk scores - Novel macromolecular structure - Gene expression microarray data (must be MIAME compliant) - Crystallographic data for small molecules - Mass spectrometry-based proteomics data   For more information on mandatory data deposition policies at the Nature Portfolio, please visit <http://www.nature.com/authors/policies/availability.html#data>  For an up-to-date list of approved repositories for each mandatory data type, please visit <https://www.springernature.com/gp/authors/research-data-policy/repositories/12327124>.  **Accession code(s) for deposited data must be provided in the Data Availability statement in the final version of the paper.** Failure to do so will delay publication. Please ensure data are available prior to publication. |  |
| *Communications Biology* has a strong preference for all data to be deposited in an approved repository. In some cases, data deposition may be required by the editor.  **We recommend the following data repositories:**   - [GenBank](https://www.ncbi.nlm.nih.gov/genbank/) (all DNA sequence data) - [NHGRI-EBI GWAS Catalog](https://www.ebi.ac.uk/gwas/) (GWAS summary statistics) - [PGS Catalog](https://www.pgscatalog.org/) (polygenic risk scores) - [Gene Expression Omnibus](https://www.ncbi.nlm.nih.gov/geo/) (Microarray or RNA sequencing data) - [Sequence Read Archive](https://www.ncbi.nlm.nih.gov/sra) (WGS or WES data) - [Protein Data Bank](https://www.wwpdb.org/) (protein structural data) - [OSF](https://osf.io/) (neuroimaging raw data and EEG/EMG/MEG raw data) - [Neurovault](https://neurovault.org/) (unthresholded statistical maps, parcellations, and atlases produced by MRI and PET studies) - [Image Data Resource](https://idr.openmicroscopy.org/about/) (microscopy data) - [PRIDE](https://www.ebi.ac.uk/pride/) (proteomics data)   Data types without a specific repository can be deposited in a generalist repository, such as [figshare](https://figshare.com/) or [Dryad](https://datadryad.org/stash).  For an up-to-date list of approved repositories, please visit <https://www.springernature.com/gp/authors/research-data-policy/repositories/12327124>. |  |
| **Data citation**  Please cite datasets stored in external repositories **in the main reference list**.  For previously published datasets, we ask authors to cite both the related research articles and the datasets themselves.  For more information on how to cite datasets in submitted manuscripts, please see our [data availability statements and data citations policy](https://www.nature.com/documents/nr-data-availability-statements-data-citations.pdf). |  |
| **Code availability**  Please include a Code Availability statement, indicating whether and how the code can be accessed, including any restrictions to access. **In some cases, the editor may require that code be made immediately available.**  This section should also include information on the versions of any software used, if relevant, and any specific variables or parameters used to generate, test, or process the current dataset. **The Code Availability statement must be provided as a separate section after the Data Availability section.**   Please see our policy on code availability for more information. <http://www.nature.com/sdata/for-authors/editorial-and-publishing-policies#code-avail>  In addition to making the custom code available, please ensure that the version of the code/software described in the paper is **deposited in a DOI-minting repository** (eg, Zenodo) and that this DOI is also cited in the main Reference list. | Please move the information on codes (GO plot etc) in the CAS here.  A Code Availability statement was included:  **Code availability**  The pipelines used for in situ sequencing analysis are available at <https://github.com/Moldia>.  GOplots were created using the R package available via CRAN-The Comprehensive R Archive Network: http://cran.r-project.org/web/packages/GOplot). |
| **End Notes** | |
| Please check that your bibliography complies with the following:   - Your bibliography should start with the heading “References”. The references must be numbered in the order of appearance in the text, then tables, then figures. - Any in-text citations to references (e.g. "Gupta et al. show...") should be followed by their corresponding reference citation number from the reference list. - Manuscript citations must include journal title, article title, volume number, page or article number or DOI, and year of publication. - No publication can be present more than once in the reference list. - No footnotes are permitted in the references or elsewhere. Text should be incorporated into the main text, the Methods section, or the Supplementary Information instead. - Websites should only be listed in the references if they are in common use or curated. - Where possible, preprints in the reference list should be updated with details of the published, peer-reviewed paper. - Citations should be formatted in the text using superscript numbers. |  |
